# Abstracts &c.

**Published:** 1889-03

**Authors:** 


### ABSTRACTS AND GLEANINGS.

**Sulfanol--the new Hypnotic.**—*N. Y. Med, Record*, Oct. 6:—The main facts regarding sulfanol are already well known; the articles of Kast, its discoverer; of Rabbas, of Schwalbe and of Wendt, have informed us that it bears the beautiful chemical designation diaelthylsulfondimethylmethan; that it belongs to the group of disulfons; that in doses of 1.0 to 3.0 or 4.0 it has a decidedly hypnotic action, and that it differs from other hypnotics, not to say narcotics, in restoring the natural desire for sleep. The experience of the German authors mentioned above has been highly satisfactory, even Schwalbe obtaining good results in sixty-six per cent. of his cases.

My own experience with sulfanol is limited to about sixty trials on fifteen patients. These cases include a considerable variety of troubles, and a fair number of those troublesome cases of functional insomnia on the treatment of which I wrote a short article about a year and a half ago. Limited as my experience has been, it is sefficient to show that this new hypnotic is a valuable addition to the Pharmacopœia, but that it is not infallible; and what drug is? The drug was administered, as a rule, in 2.0 doses, to be taken one-half hour before bed-time,

Clinical histories of ten cases are given. Conclusions are:

1. These cases prove that sulfanol is valuable in cases of functional (neurasthenic) insomnia. In those cases in which insomnia is often the most distressing symptom, it appears to justify the claim of its discoverer that it restores the natural desire for sleep, two grammes being sufficient to bring about this result.

2. It is easily tolerated by the stomach; does not produce headache, except in large doses of 3.0 and more; has no effect upon the circulation, and can be given in cases of irritable and fatty heart, and apparently in cases of fever, in all of which respects it appears to be superior to other hypnotics.

3. It has very slight or no narcotic virtues.
4. With some patients the drug appears to lose its effect if frequently administered.

If a larger experience than I have had will prove sulfanol to be a safe hypnotic in fever cases, all medical men will recognize it as a great boon. In the treatment of insomnia accompanying mental troubles its success is assured; in the treatment of neurasthenic insomnia it promises very well indeed, and the treatment of this trouble can be inaugurated by the exhibition of this drug.—DR. SACHS, IN *N. Y. Med. Record.*

**The Arsenite of Copper in Cholera Morbus.**—Some time ago Dr. Boardman Reed, of Atlantic City, began the use of the arsenite of copper for the relief of such intestinal affections as are usually found amongst those who remove to a new locality, involving a change of air, food and water, the surroundings of that resort being equally beneficial to persons in winter as well as summer. He says: "My experience is that $\frac{1}{1000}$ grain of cuprum arsenite, given in divided doses every quarter hour, so that the whole amount may be taken in the course of three or four hours, will rapidly cure most cases of cramps or colic, especially if associated with diarrhœa, some relief usually being experienced within the first hour. It suits all the better when vomiting as well as diarrhœa accompanies the cramps—in other words, the typical case of cholera morbus. Of course, my explanation of the effect is that the small doses used act in exactly the contrary manner from large or physiological doses of the same drug.

"At the suggestion of Dr. Reed, I procured a sample of this remedy in the latter part of July, and during the succeeding two weeks it was used in probably twenty cases of bowel troubles, in patients ranging from one year up to sixty or more, and varying from simple colicky pains to diarrhœa and vomiting of several days' duration, and one case of acute dysentery accompanied by profuse bloody discharges from the bowels, and in every instance the treatment proved eminently successful. Not a single failure occurred, and as a rule, the pain and tenesmus subsided after the first hour, or after taking of the first five doses. At an expense of ten cents I purchased a trituration of the remedy of the strength of one part to 1,000 parts of sugar or milk, each patient being provided with a quantity estimated at one grain, which was dissolved in about half a glass of water—four to five ounces—the directions being to take a teaspoonful every ten minutes for the first hour, then hourly or half-hourly thereafter, according to the urgency of the symptoms. The dose for children, therefore, is the same as that for adults, as near as can be calculated, $\frac{1}{40000}$ of a grain. These small doses accord with my views, and the happy effects attending this method have proven exceptionally interesting and satisfactory.—*Med. Register—Epitome.*

**Strychnia an antidote for Morphine Poisoning**—*Sou. Pract.*, October:—Mr. C. took at 4 o'clock, P. M., while drinking, six grains of morphine, and in a short time was sound asleep and could not be aroused. In half an hour afterwards an effort was made to clear the stomach by vomiting, without effect, and all other means to arouse the young man proved as futile as the emitics; the man slept in spite of everything. I saw him at 8 o'clock p. m., four hours after he took the morphine and found him with all the symptoms of morphine poisoning, pulse slow, and breathing very slow and stertorous. In fact, he was in a profound coma, and it was concluded by all that he must die. His extremities were cold and blue, no muscular resistance could be excited by any means. I gave him one-thirtieth of a grain of strychnia hypodermically, and in a short time his pulse and breathing improved greatly, and in ten or fifteen minutes he opened his eyes when violently shaken and sharply spoken to. At this time efforts were made to awake him, after which time, say about one hour, I again injected him with one-thirtieth of a grain of strychnia, and in a short time was surprised to see the magical effect of the remedy. In a few minutes more the man was conscious. During the interval between the injection of strychnia he took five grains of carbonate of ammonia every half hour. He made a good recovery.—DR. FLETCHER IN *Southern Prvctitioner*.

**The Action of Chlorofrm-Water as an Antiseptic and Germicide.**—In the *Deutsche Medicinsche Wochenschrift*, No. 16, 1888, Salkowski reports the results of studies which he has made upon this subject. It has been common stock knowledge with most of the profession for a number of years, that chloroform water added to urine prevents its decomposition, and, also, that chloroform prevents all those fermentations which depend upon the growth of micro-organisms, as for example, the fermentation of yeast, the ammoniacal fermentation of urea, and the conversion of hippuric acid by fermentation into benzoic acid and glycocol. It is also to be remembered that chloroform has in reality no power in the prevention of fermentation, the result of the action of such ferments as ptyalin and pepsin.

Salkowski has found that by the addition of a little chloroform, milk may be kept in a well corked bottle for months, keeping its alkaline reaction and finally changing to a jelly which upon shaking, separates into two layers, one of which consists of a clear yellowish fluid, the other being made up of casein and fat. Meissner has attempted to explain this gelatinous change, which occurs in sterilized milk, as being due to the action of a slowly acting milk, curdling ferment. It has also found that both cane and grape sugar do not ferment, when yeast is added to them, provided chloroform is present, and the still more interesting fact is noted that nevertheless in twenty-four hours cane sugar is changed to invert sugar by some unknown ferment from the yeast.

Salkowski has also determined that albuminous liquids and meat will remain free from organisms if in the presence of chloroform, and that under these circumstances no growths can be developed on nutrient media.

It is also of interest to learn that chloroform not only prevents the growth of micro-organisms, but also destroys them even though their development is extraordinary, for, if it be added to putrifying beef tea or applied to silk threads impregnated with anthrax bacilli, both rapidly become innocuous, the beef tea being sterile at the end of half an hour, and the anthrax bacilli becoming innocuous after twenty-four hours; again, crushed spleens from animals suffering from splenic fever are rendered completely innocuous after thirty minutes exposure, and the comma bacillus of a fresh cholera cultivation, mixed with an equal volume of chloroform water becomes sterile in sixty seconds. It was also found that when guinea pigs were innoculated with half a Pravaz's syringe full of fluid, composed of one drop of anthrax blood and eight cubic centimeters of sterilized water, death ensued in the course of two days, whereas in those instances where chloroform water was used with anthrax blood the animals remained healthy.

There are several practical advantages to be gained by the physician from this research, for it is proposed that a few drops of chloroform may be added when preparing solutions for subcutaneous injection which readily undergo putrefaction, and from which the chloroform may be driven by heat, or exposure to the air, before the solution is used; and also to employ it internally in those diseases of the digestive organs dependent upon the presence of micro-organisms, particularly in the stomach. It is a useful and cheap preservative for anatomical preparations. As Salkowsky has administered to dogs, weighing 36.8 kilos no less than 200 cubic centimetres, or nearly 7 ounces of chloroform-water with the food for four days without the production of any symptoms, it would seem probable that its internal use in man is without any danger. It has been proposed that chloroform water should be added to milk or other foods during artificial digestion when the process is to be stopped in order to avoid the development of the bitter taste. As, however, chloroform does not act upon digestive ferments, this use of it would seem to be theoretically and practically impossible.—*University Med. Magazine.*

**On Some Peculiar Effects of Circumcision.**—(*Deutsche Med. Zeit. October 8, 1888, Révue Méd. de la Suisse Romaine.*) .Professor Reverdin was forcibly impressed by a brochure of an American colleague who claimed that circumcision was indicated in bed-wetting, and that it often relieves patients of coxalgia. After trying it he gives his experience. A boy nine years old, and well developed, complained for several weeks of severe pain in the right hip, and walked lame. On examination in the upright position nothing abnormal was found; when he walked the mobility of the affected limb was diminished. If force was used in moving the leg severe pains were felt and the muscles were strongly contracted. The pains radiated through the iliac fossa, the upper part of the thigh, and the inside of the arms. Measurement gave no definite results. The case seemed to be one of beginning hip-joint disease. ℞ ordered rest and extension. In spite of this treatment, for half a year, there was no improvement, there was no swelling or sign of suppuration, no difference in measurement, but the same functional disturbance and severe pain. At the last examination the great length and narrowness of the prepuce was noticed, although the patient had never had any symptoms referable to that, nevertheless circumcision was performed and all the above-mentioned symptoms disappeared as if "before the wand of a magician."

Another case was still more strange and inexplicable. A young man became morose, melancholy, apethetic, and showed a decided suicidal tendency. His disposition changed completely and satisfactorily after circumcision. Reverdin does not pretend to explain the causal connection between the psychical change and the operation although he says there was nothing in the patient's external relations to bring about the result. Fleury published similar cases of the cure of hysteria and hypochrondria by circumcision. In the practice of Professor J. L. Reverdin, his cousin, were the notes of two cases when epileptic convulsions were perminently cured by the removal of a long, narrow prepuce.—*American Practitioner and News.*

**Hydriodic Acid.—Its Uses in General Practice.**—Dr. W. C. Wile, of Connecticut, believes that the advantages which this remedy possesses over other forms of iodine are not sufficiently understood, nor as thoroughly appreciated as they should be. It is his favorite remedy in all asthmatic troubles, which generally yield promptly and effectually to it. It also produces most excellent results in cases of chronic bronchitis of long standing, and in such he has often observed that small doses, frequently repeated, are often of signal service, when larger doses do not seem to accomplish the same results. It has yielded very satisfactory and often surprising results in all forms of chronic lead poisoning, combined with saline cathartics and the Faradic current. Excellent effects have also been obtained from its use in scrofulous diseases, especially of children. He is now using it in a case of obesity with the result of a steady diminution of the amount of fat without a single disagreeable symptom, or interference with the general health, or the action of any of the functions of the body. It has yielded its most magnificent and rapid results in all the latest stages and manifestations of syphilis. The general plan of administration is to begin with small doses of Gardiner's syrup, fifteen drops, and gradually increase each dose by a drop until the point of toleration is reached.—*New Eng. Med. Monthly, August, 1888.*

**The use of Ethylate of Sodium in the Treatment of Lupus.**—In the *British Medical Journal* for October 6, 1888, Mr. L. A. Taylor reports the case of a woman, aged 58, who had suffered from lupus of the face for fifty-four years. During this time no local means for removing this appeared to be adopted. The lower side of the face was covered with cicatricial tissue and the lower eyelid was everted. The surface over the masseter muscle down to the angle of the jaw was covered by a patch of lupus exedens. The whole of the nose was similarly affected, and there was another patch above the inner canthus of the same side. After chloroform was given, Mr. Taylor scraped the lupoid surfaces with a small oval-shaped Volkmann's spoon, taking care to thoroughly remove the whole of the diseased tissue, and then applied nitric acid with a glass rod. The wounds healed in a fortnight. After a little time, however, several small patches appeared, one above the inner canthus, one over the prominence of the right malar bone, another immediately in front of the ear, and others on the left side of the nose, on the right half of the upper lip and immediately external to the right angle of the mouth. An anæsthetic was again administered on June 28, and these patches treated as in the first operation. These soon got well, with the exception of the patches near the mouth, which were prevented from healing by the constant movements of the lips. Shortly after this second scraping several small patches appeared, and in the course of a day or two grew so rapidly that every time the patient was seen they were much larger. Mr. Taylor then decided to treat them with the liquor sodii ethylatis. This was applied to the patches with a glass tube, which had a very small bore, once a day for three consecutive days. Two or three days after the last application of the solution the patches were covered with dry scabs, which either fell off themselves or were easily removed with a pair of dissecting forceps, leaving a perfectly healthy surface beneath. Since this time other small patches have appeared, and these have been treated in a similar manner. The treatment has been so successful that the appearance of the patient is vastly improved. She is anxious now to have an operation for the ectropion performed.

The advantages of using the ethylate of sodium solution in the treatment of small patches of lupus are that it is not necessary to give an anæsthetic, and so the idea of having an "operation" performed is removed from the patient's mind. It only takes a minute or two to apply the solution; a very slight tingling is produced, which passes away in three or four minutes. After the removal of the scab there is no wound left to heal, and consequently there is no necessity for the patient to walk about with a dressing on the face. In this case the recurrence of fresh places was rapid after the second scraping operation, while the new patches which have appeared during the time the ethylate of sodium treatment has been carried out have been small, and have grown slowly. During the time the solution is being applied, no water should be allowed to touch the parts.—*Therap. Gaz.*

**Night-Terror and Screaming in a Child Cured by Removal of the Tonsils.**—Dr. J. M. Elborough Scatliff reports in the *Lancet* for October 6, 1888, a case of a boy about 7 years of age, to all appearances in good health, who seemed to be quite well all day, took his food with appetite, and had good spirits; but every night after he had been asleep some little time he used to wake up in a state of great terror and alarm, cried out and refused to be comforted. In a short time he got over the attacks and became composed and rational, and would lie down quietly to sleep again. Dr. Scatliff was unable to find anything whatever the matter on examination of the boy with the exception of greatly enlarged hypertrophied tonsils. Having decided that these were the cause of the symptoms, he removed them both with very little hemorrhage. The result was that the little patient entirely got rid of his night-terror and screaming. Dr. Scatliff's premise seems warrantable that in deep sleep when he lay in some unfavorable position the tonsils obstructed the respiration so as to cause imperfect aeration of the blood and the disturbed mental condition.—*Therapeutic Gazette.*

**Digitalis In Remittent Fever.**—The peculiar climate conditions of this country seem to favor the varous remittent forms of fever, more especially the well known and justly dreaded "Typho-Malarial."

An active practice of nine years has given me ample opportunity to test most of the prevalent modes of treatment of such affections, all of which seem more or less unsatisfactory; so much so, indeed, that a majority of our best physicians have settleed quitely down to tha expectant plan. Two years ago I began to notice that when, from cardiac weakness or other causes. I had occasion to use digitalis with any regularity the unfavorable symptoms decreased steadily after thirty-six hours aud the fever terminated in a few days. Continuing my investigations I discovered that the fluctuations of the febrile movement were greatly lessened under the influence of this drug and have seldom noticed a greater variation than two degrees in twenty four hours. I became so thoroughly convinced of the utility of the drug that during the year 1888 I resorted to the foxglove on the first appearance of typhoid symptoms, with the uniform result of entire subsidence of fever in five days from commencing the digitalis. I treated sixty-nine cases of true typhoid and typo-malarial cases without the loss of a single patient, and, as a rule, the fever subsided on on the fifth day of treatment with digitalis.

Dr. G. A. TROTT, in *Med Age.*

**Pichi In Cystitis.**—I have used pichi in several cases of chronic catarrh of the bladder with muco-purulent discharges, and find it beneficial to this extent: The discharge was made more fluid and consequently easier to pass; the ejaculatory muscles seemed to be toned up after having in some cases, been weakened almost to paralysis, particularly in an old man of seventy-five years, who had a catarrh of the bladder and enlarged prostate gland of over ten years' duration. I was led to the use of the remedy by the claim of its introducer that it was a "Lithontriptic" and that it acted upon the mucin by which the siliceous matter in stone was held together. I reasoned that if it did not act to this extent, it might to the extent of dissolving and rendering more fluid the viscid discharge. To this end I gave it and think I have had satisfactory results. The old man's case was an interesting one and at some future time will be reported. I have not discovered that the urine has been materially increased.

Dr. CHAS. E. NASH, in *Med. Age.*

**Treatment of Sprains.**—DR. C. A. WEST (*Chicago Medical Times*:) Every doctor has been perplexed with the treatment of sprained ankles or wrists or knees. The treatment must often be prolonged, and the pain and swelling often remain for a long time, until the patient, who is apt to be an active, restless, healthy business man, becomes no longer patient, or your patient, as he consults some other medical man, who may inform him that his great mistake was in not consulting him at first, as all valuable (?) measures have been neglected.

Liniments in these cases are of but little use. Relief from pain is the first essential to be procured in a way which will further the process of cure. This may be done by stimulating the circulation of the part, thus preventing blood stasis and engorgement about the part. Immerse the injured joint in hot water, for from twelve to eighteen hours if necessary. As soon as the major portion of the swelling and the pain has been abated, apply to the afflicted part a light plaster of Paris or starch dressing to insure immobility, and be assured that the cure in most cases will be very speedy and remarkably satisfactory. The writer has tried this in several cases, and he has yet to have a single unsatisfactory result. The only remedy necessary, if any be used, is arnica, diluted with five parts of water or sweet milk applied for a few hours before the permanent dressing.

**Chronic Ulcer of the Leg.**—By J. W. Forney, M. D., Sunbury, Pa. *Med. Register, Nov. 3.* The writer illustrates his views on treatment by the narration of the following case: Henry H. aged sixty-four, bachelor, was first seen by me in June, 1878, for a large and troublesome ulcer of the right leg, three inches above the ankle. It was as large as the top of a coffee cup. Its general surface was depressed, the edges dry and elevated, irregular and dark colored. The floor, which rested upon the tendons of the muscles beneath, was anything but "pyogenic," for it had for a long time been generating nothing but a thin, icherous liquid from an unhealthy, grayish, glistening surface, void of any pretension of granulation. There was not a single favorable feature about the case. The patient was old, and the sore was of twenty years' standing. The hygenic conditions were very bad as to cleanliness, food and general habits. He had but recently recovered from an attack of remittent fever, which had considerably reduced his powers of recuperation. He had reached a stage of resignation due to having been told by a physician that it would be certain death to have the "habitual discharge" stopped. I had attended him through his last sickness, which he had expected would be his end, and the consequent confidence led him to believe when I told him his sore leg could be made well.

The ulcer was dressed as follows: From an old muslin piece I first cut a patch the exact size and shape of the sore inside the raised border. I then trimmed a piece of oil silk to the same shape, but so as to extend one-third of an inch beyond the edges of the muslin. The muslin was then dipped in a solution of corrosive sublimate (two grains to three ounces of water) and applied to the depressed surface of the sore, nicely fitting within the edges. Oiled silk was then adjusted over the top of this application, its edges extending to the line of healthy tissue. Over the limb, from the toes to the knee, was applied a roller bandage. This dressing was changed once or twice daily as long as the corrosive sublimate solution was used. From the very first an improvement was apparent. The glistening, pale surface of the ulcer began to redden, and to have poured out a better quality of pus. The hardened margins softened and slonghed away to healthy adjacent tissues. This treatment required about two weeks. I had now instituted what I desired—action—and henceforth I expected to continue the cure with no trouble. Discontinuing the corrosive sublimate solution I substituted for it a solution of sulphate of zinc (one half grain to the ounce of water.) As the process of repair went favorably on, if anything led me to suspect hyper stimulation, simple water dressing was used for a few times. As little, healthy granulations, which in turn became larger and progressed towards the centre of the ulcer notches, corresponding to the granulations in size, shape and position were made in the muslin so as not to include them in its covering. The oiled silk was trimmed accordingly as soon as the new growths had attained sufficient size and maturity. The more mature they became the stronger seemed their disposition to push out into and to fill up the unhealed portion of the ulcer.

The old gentleman lived for eight years, comparatively free from sickness, dying about a year ago of a fever.—*Epitome.*

**Climatic Influence on the Morals.**—Modern French scientists are nothing if not methodical, and have repeatedly called attention to the curious regularity in the geographical distribution of certain vices and virtues: Intemperance, for instance, north of the forty-eight parallel; sexual aberrations, south of the forty-fifth; financial extravagance in large seaport towns; thrift in pastoral highland regions. It is, indeed, a remarkable circumstance that in the home of the best wine-grapes, in Greece and southern Spain, drunkenness is far less prevalent than in Scotland, or in Russian Poland, where Bacchus can tempt his votaries only with nauseous vodka. The idea that a low temperament begets an instinctive craving for alcoholic tonics seems disproved by the teetotalism of the Patagonian savages, who horsewhip every Spanish stimulant-monger without benefit of clergy. The Lesghian mountaineers, too, observe the interdict of the Koran in the icy summit regions of the Caucasus, but there is no doubt that the bracing influence of a cold climate affords a certain degree of immunity from the debilitating effect of the alcoholic vice, and that the Scandinavian peasant can for years survive the effects of a daily dose of alcohol that would kill an Egyptian fellah in a single month. But it is equally certain that the têmperance of Southland nations is considerably facilitated by the abundance of non-alcoholic pastimes. The Spaniards have their fandangos and bull fights, the Greeks their border raids, cock mains and horse races, while the Scotchman, after six days of hard work is confronted with the choice between the delirium of alcoholic fever and the appaling tedium of Sabbatarian ascetism, and naturally chooses the less dismal alternative.—*Dr. Oswold in Pop. Sci. Monthly.*

**Lanolin and Boric Acid in Skin Diseases in children.**—The combination of lanolin and boric acid as an ointment is said to have a most gratifying effect in certain skin diseases in children, especially eczema of the head and face, intertrigo, and seborrhœa. In the case of eczema, for example, with raw patches on the cheeks and yellowish crusts on the head, the surface is first cleansed in the usual way, and then dusted over with finely powdered boric acid. On the following day this washing and dusting over is repeated; already the inflammation will seem lessened. The process is then repeated twice daily, the washing always being done gently, until the skin is in a condition to bear an ointment containing thirty per cent. of lanolin and eight per cent. of boric acid. In the squamous form of eczema with considerable induration, olive oil is well rubbed in, and then removed with castile soap, and an ointment containing one-half or one per cent. of salicylic acid with thirty per cent. of lanolin is encrgetically applied according to the degree of indurition. This washing and application are repeated twice daily. The strikingly beneficial action of this course of treatment, which is less painful than the use of strong alkalies or oil of cade, is ascribed to the penetrating properties of lanolin, which thus facilitates the entrance of salicylic acid into the deeper layer of the epidermis. Dr. Russell Sturgis, who advocates the above treatment, also finds lanolin a reliable means of alleviating the irritation due to chronic uticaria.—*British Med. Jour.*

**Treatment of Incontinənce of Urine by Rhus Aromatica.**—Rhus aromatica has recently come into vogue in France as a remedy for incontinence of urine in atonic states of the bladder. According to *La Thérrapeutique Contemporaine*, Drs. Max, Burvenich, Numa and Hamon have all tried it in this disorder, and have obtained signal successes where all other remedies have failed.

Max prepares a tincture by macerating two hundred grammes of the bark in a thousand grammes of alcohol at 80°. The dose is from 10 to 15 drops three times a day. Max claims nine cures out of eleven cases. Burvenich obtained a cure in eleven out of thirty-three cases; ten of the remaining twenty-two were considerably benefited. The good effect of the medicine is not felt till the fifth or sixth day; sometimes satisfactory results are not perceived till at the end of three or four weeks.

According to Burvenich, Rhus aromatica constitutes a powerful tonic similar to nux vomica, for in a man aged 79 years, affected with incomplete paralysis of the bladder without stricture of the urethra or hypertrophy of the prostate, Rhus aromatica, in the dose of 30 drops a day, greatly facilitated micturition by giving tone to the bladder. Numa also asserts that it excites the unstriped muscle of the bladder, as well as of the uterus and the rectum. He gives the tincture to children from 2 to 6 years of age in the dose of 10 drops twice a day, and to older children in the dose of fifteen drops night and morning. The tonic effects do not last, for the paresis of the sphincter vesacæ muscle returns when the medicine is discontinued.

Hamon has also testified to the efficacy of Rhus toxicodendron in certain cases of incontinence of urine, with atony, in young persons. He gives 20 drops at bedtime in a little simple elixir, or other convenient menstruum. This use of Rhus toxicodendron is not new.

The Rhus aromatica is the *fragrant sumach*, which is indigenous in this country, growing in dry rocky soil from Vermont to Michigan, Kentucky, and westward. It is a low, straggling bush, with sweet-scented leaves. The Rhus toxicodendron is the well-known *poison ivy.—Boston Medical and Surgical Journal.*

**Inflammatory Sore Eyes.**—The general practitioner is often at a great loss for data on diseases of the eye, and reference to works on general practice shows them to be lacking in detail and often of little assistance, hence, I submit this paper to the numerous readers of your esteemed journal. Such cases are of frequent occurrence with the general practitioner, and are often as annoying as frequent, but which may be successfully treated by the methods and remedies exhibited in the following reports. The cases will be classified under the four forms of conjunctivitis, viz.: *Simple, Catarrhal, Purulent and Pustular.*

*Simple Conjunctivitis.*—Miss C., aged 16 years. She has read a great deal in the cars, and by bright light. Eyes present a general appearance of irritation and conjestion, red, tender, painful, conjunctiva hyperæmic.

Treatment:

| | | |
|---|---|---|
| ℞ | Tannin . . . . . . . | grs. v. |
| | Sodii biborat . . . . . . | " x. |
| | Glycerin . . . . . . . | ʒ i. |
| | Aq. purœ . . . . . . . | o. i. |

M. Sig. Spray the eyes three times a day, and apply cold wet cloths over the eyes for one hour, four times a day.

Also:

℞ Tr. Bellad.

Dose—Drops two; four times a day.

Case made steady improvement and was discharged cured in three days.

Case 2—Mr. D., aged 45 years, farmer, has been troubled with inflammatory sore eyes for several years. Examination revealed *granular lids.*

Treatment:

| | | |
|---|---|---|
| ℞ | Ferri Sulph. . . . . . . . | grs. x. |
| | Ac. Sulph. Arom. . . . . . . | gtt. i. |
| | Aq. Dist. . . . . . . . | ℥ i. |

M. Sig. Apply with camel hair brush once daily over the granulations, everting the lids. Discharged cured at the end of two weeks. This is my favorite application for granular lids and has often won for me the gratitude and patronage of many families. It is applicable in all inflammatory conditions of the conjunctiva when associated with granulations.

**Catarrhal Conjunctivitis.**—Case 1. Mr. L., aged35, a carriage painter, was several days under treatment before seen by me. Right eye very much inflamed, watery discharge, great pain, photophobia, severe chemosis, so that the pupil was almost hid from view when lids forcibly opened, an ulcer was located on the cornea; both upper and lower lids much swollen; frontal headache; patient very nervous.

Treatment:

| | | |
|---|---|---|
| ℞ | Cupri Sulph. . . . . . . | grs. v. |
| | Aq. Dest. . . . . . . | ℥ i. |

M. Apply lightly to the ulcer under cocaine, once daily.

| | | |
|---|---|---|
| ℞ | Atropin Sulph. . . . . . . | gr. i. |
| | Ferri Sulph. . . . . . . | grs. v. |
| | Ac. Sulph. Arom. . . . . . . | gtt. i. |
| | Aq. Dest. . . . . . . | ℥ i. |

M. Apply to the affected conjunctival surface, including the chemosis, three times a day.

| | | |
|---|---|---|
| ℞ | Ammon Brom. . . . . . . | ℥ ss. |
| | Tr. op. deod. . . . . . . | ʒ ii. |
| | Elix. Simp. . . . . . . | ℥ ss. |
| | Aq. ad. . . . . . . . | ℥ ii. |

M. Teaspoonful in water every three hours.

Patient steadily improved and was discharged cured in one week, at which time he resumed work.—*Western Med. Reporter.*

**Purulent Conjunctivitis.**—Case 1. Mr. H. Patient first complained of itching, and a sensation of sand in the eye, followed by smarting and burning and discharge of a thin, acrid fluid from the affected eye. The secretion soon became thicker and purulent, swelling of the lids and chemosis occurred. At this stage of the disease the patient was seen. Examination revealed an alarming condition of purulent ophthalmia, the chemosis being extreme and extending to the lids, the cornea being almost buried behind the chemosis, there being free effusion into the areolar tissue; pain in the orbit and frontal sinuses, the eye injected with blood. I treated this case as I do all cases of purulent ophthalmia whether *simple or specific.* In this disease, which is the most rapidly destructive disease of the eye, cleanliness is better than Godliness. All discharge was washed off, rubbing all thick matter off with absorbent cotton, and thoroughly cleansed the eye with a 5 per cent solution boracic acid, then applied the following ointment:

| | | |
|---|---|---|
| ℞ | Atropin Sulph. . . . . . . | gr. i. |
| | Hydrg. ox. flava, rubbed very fine. . . | grs. xvi. |
| | Boracic acid. . . . . . . . | grs. xx. |
| | Cocaine Mur. . . . . . . | grs. x. |
| | Ung. Petrol. . . . . . . | ℥ i. |

M. This was applied thoroughly and freely to the whole conjunctival surface, and then the eye was covered with lint smeared with the same ointment, and then a bandage applied. As the first application of a two per cent solution cocaine, but this is not afterward needed.

The eye was examined in two hours and as there was some discharge the process was repeated. After this the eye was ordered dressed and ointment applied every four hours, no discharge being allowed to accumulate at any time. Aconite and opium were given internally. Improvement began at once and progressed to a favorable termination in five days. There were no structural injuries to the eye, and it was left as well as ever.—*Ib.*

**Amputation of the Pregnant Uterus.**—Mr. Lawson Tait, who has greatly improved the technique of this operation, says that it is the easiest operation in abdominal surgery, and every country practitioner ought to be able and always prepared to do it. He predicts that it will revolutionize the obstetric art, and in a short time we shall hear no more of craniotomy and eviscerations; for this new method will save more lives than these proceedings do, and it is far easier of performance. He describes the operation as follows:

No special instruments are required, nothing but a knife, some artery forceps, a piece of rubber drainage tube, two or three knitting needles, and a little perchloride of iron. My method of operating is to make an incision through the middle line, large enough to admit my hand, and then I pass a piece of rubber drainage-tube (without any holes in it) as a loop over the fundus uteri and bring it down so as to encircle the cervix, taking care that it does not include a loop of intestine. I then make a single hitch and draw it tight round the cervix, so as to completely stop the circulation. I give the ends of the tube to an assistant, who keeps them well on the strain, so as to prevent the loose knot from slipping, the reason for this being that should there be any bleeding and any necessity for further constriction, I could secure this in a moment without undoing any knot; and the simplicity of this method greatly commends it. I then make a small opening in the uterus and enlarge it by tearing with my two forefingers, seize the child by the foot and remove it. I then remove the placenta, and by that time the uterus has completely contracted and is easily drawn through the wound in the abdominal wall. The constricting tube will now probably require to be tightened, and the second hitch of the knot may be put on at the same time, and the work is practically done. Stuff a few sponges in the wound to keep the cavity clear of blood, and pass the knitting-needles through the flattened tube and through the cervix, and in this simple way a clamp of the most efficient kind is at once made; the uterus is removed about three fourths of an inch above the rubber tube. The usual stitches are put in, the wound closed round the stump, which of course is brought to the lower part of the opening, and then the stump is dressed with perchloride of iron in the usual way. The operation takes far less time to perform than it takes to describe, and as there is hardly any possibility of complications, it is one of the simplest operations that can be undertaken, and must always be pretty much the same; for this reason no one need ever be in any fear about undertaking it, for the absence of variation in the difficulties to be encountered, it differs entirely from any other operation in abdominal surgery.

If performed before the patient has been mauled about by ineffectual attempts to deliver, its mortality will be no greater than that of ovariotomy, and the arguments in its favor against all alternative proceedings are, first, that it can not be more dangerous to the mother than most of these are; that it saves the life of the child; that it prevents the unfortunate mother from again being placed in a similar condition; and it certainly has the great advantage over alternative proceedings having a similar object; that its great simplicity, as contrasted, for instance, with operations proposed by Thomas, Muller and Sanger, will make it possible for the country doctor, less experienced in surgery, to perform it without hesitation. These complicated and difficult proceedings may have their advantages, though I confess I do not see them; but they will be left for the hands of experienced specialists. The operation I have described will be the operation of emergency, where only the resources of general practice are at hand.—*Indiana Medical Journal.*

**A new Antiseptic Surgical Dressing.**—By H. Bendeblack Hewetson, M. R. C. S. *Northwestern Lancet,* China grass is a soft, silky, very highly absorbent fibre used in various manufactures. The combings form an elastic silken wool, which when treated with four per cent of salicylic acid provides an excellent antiseptic absorbent surgical dressing. It is also relatively much cheaper than so far as I know, any of the usual dressings used in surgery. I have not given it an extended trial, and supported by the opinions of most of my medical friends have ventured to bring it before the notice of the meeting of the Leeds and West Riding Medico-Chirurgical Society. I am indebted to Messrs. Reynolds and Branson of Leeds for the admirable way in which the raw material is treated and prepared as an antiseptic dressing, of whom it may now be purchased. Its chief value is the way in which it absorbs discharges from a wound, rendering it very dry, and preventing the poulticing of the wound, as it were, when bathed in discharges under less absorbent materials. A considerable quantity is required in cases where the discharges are free, and it is perhaps well to interpose some open meshed gauze between the dressing and the wound so as to prevent the China grass from sticking to the wound when removed. I have also observed that under the pressure of bandaging the material still retains its absorbing qualities, and does not cake when properly teased out before use.

**Disinfection of Surgeons' Hands.**—A great deal has recently been said about surgeons' nails and nail-brushes in hospital wards. Bacteriologists have come to the conclusion that the usual washing of the hands with weak antiseptics is quite efficacious in killing pyogenic and specific germs on the hands, but does not destroy micro-organisms which lodge under the nails. Dr. Furbringer, of Berlin, believes in washing the hands with soap, then in alcohol at 80° F., then in a sublimate solution. The alcohol is said to soak well into the nails. Drs. Roux and Reynes have tried experiments with alcohol on nails purposely infected. In forty cases where the infection was experimental, and carried on in a laboratory, asepsis was proved in thirty-three. In eight cases, where infection was clinical, the hands being washed in the usual manner after touching infected fluids in wards only four were proved aseptic after the above elaborate system of washing. Drs. Roux and Raynes nevertheless advocate this antiseptic ablution, noting that the nails are seldom so thoroughly rubbed after every operation as when an experienced physiologist cleans them after some special experiment. In all cases of enforced sanitary measures, routine practices and habits must be taken into account.—*British Medical Journal.*

**Epilepsy.**—*Antipyrin.*—M. George LeMoine, Professor *agregre* at the Faculty of Medicine at Lille, has studied the effects of antipyrin in epilepsy. He concludes that antipyrin diminishes the number of epileptic attacks, and even causes them to disappear under the following circumstances: 1. When the attacks occur at the menstrual period, and are apparently provoked by menstruation. 2. When the patients are subject to neuralgia and migraine. In every other instance M. George LeMoine believes that antipyrin produces merely transient effects.—*Brit. Med. Journal.*

**Iodine for Worms.**—By Chas. S. McKay, M. D. Lowell, Mich. *Medical Era*,: Last month I was called to attend a child about three years old, suffering from a severe cold. Noticing a discoloration on the child's chin I inquired its cause. The mother explained that about four days before the child had found a bottle of iodine (tr.) and enjoyed a taste. The mother said farther that the next day the child passed several large, round worms, the largest nearly five inches long. I had at that time on my hands a child about four years old, who had all the worm symptoms in the calendar. The worms seemed to enjoy santonine in every form, from 6x to crude drug. The day after hearing the above from the mother of the child that had taken the iodine by mistake I put a-half drachm tincture into a two drachm vial and filled it up with water, and ordered it given in three drop doses every three hours. To the satisfaction of all, the next day the child passed several round worms similar to those passed by the first babe. Another thing happened, the child had been "cankered" seemingly throughout the extent of the digestive and intestinal mucous membranes, but after taking the iodine and passing the worms, this entirely disappeared.

